# 西达本胺治疗难治性T-大颗粒淋巴细胞白血病2例临床观察

**DOI:** 10.3760/cma.j.issn.0253-2727.2023.08.016

**Published:** 2023-08

**Authors:** 小霞 李, 建平 李, 康 周, 馨 赵, 凤奎 张

**Affiliations:** 中国医学科学院血液病医院(中国医学科学院血液学研究所)，实验血液学国家重点实验室，国家血液系统疾病临床医学研究中心，细胞生态海河实验室，天津 300020 State Key Laboratory of Experimental Hematology, National Clinical Research Center for Blood Diseases, Haihe Laboratory of Cell Ecosystem, Institute of Hematology & Blood Diseases Hospital, Chinese Academy of Medical Sciences & Peking Union Medical College, Tianjin 300020, China

T-大颗粒淋巴细胞白血病（T-LGLL）是一种罕见的慢性淋巴细胞增殖性疾病，目前难治性T-LGLL尚无推荐治疗方案。西达本胺是我国自主研发的一种口服组蛋白去乙酰化酶（HDAC）抑制剂，主要针对第Ⅰ类HDAC中的1、2、3亚型和第Ⅱb类的10亚型，在治疗复发/难治外周T细胞淋巴瘤（PTCL）的初期临床研究中显示出了较好的疗效及安全性[1]–[5]。T-LGLL是PTCL的一种亚型，目前关于西达本胺治疗难治性T-LGLL尚未见报道，我们尝试应用西达本胺治疗2例难治性T-LGLL，报道如下。

## 病例资料

例1，男，53岁，因“面色苍白、乏力3年余”于2017年2月入院。3年前于当地确诊纯红细胞再生障碍（PRCA），病初血常规提示极重度贫血（HGB 32 g/L），予以环孢素、司坦唑醇治疗后，贫血一过性好转后再次下降，为进一步诊治就诊我院。患者既往糖尿病病史30余年，糖尿病肾病病史6年，高血压病史6年。入院查体：重度贫血貌，浅表淋巴结未扪及肿大，心肺未见异常，肝脾肋缘下未触及。血常规：WBC 2.67×10^9^/L，ANC 0.91×10^9^/L，淋巴细胞绝对计数（ALC）1.54×10^9^/L，HGB 50 g/L，PLT 165×10^9^/L，网织红细胞绝对计数（ARC）0.0277×10^12^/L。生化常规：Cr 130.4 µmol/L。贫血四项：血清铁蛋白（SF）497 µg/L，EPO>797.00 IU/L，血清叶酸（FA）及维生素B12（VB12）正常。免疫分型（外周血）：CD3^+^/CD8^+^/CD57^+^/TCRαβ^+^T-LGL细胞占淋巴细胞的43％，大颗粒淋巴细胞（LGL）绝对值0.66×10^9^/L。TCR重排阳性。血涂片：淋巴细胞占61％，不典型淋巴细胞易见，约占40％，胞体较大、胞质丰富、略嗜碱性、胞质中有数量不等的大嗜天青颗粒。骨髓象：增生活跃，粒系75.5％，红系 5.0％，巨核细胞大致正常，淋巴细胞占17.0％，LGL易见。染色体为正常核型。修正诊断为：T-LGLL；PRCA。于2017年2月14日起予以大剂量环磷酰胺（1.65 g，每日1次，用4 d）治疗，1周后加用他克莫司1 mg每12 h 1次口服，治疗6个月后贫血无改善。后于外院先后应用西罗莫司1 mg每日1次治疗6个月贫血无改善；硼替佐米2.5 mg每周1次×4周，观察3个月贫血无改善；艾曲波帕75 mg每日1次治疗3个月贫血无改善。患者于2019年11月再次就诊我院，查血常规：WBC 3.83×10^9^/L，ANC 2.43×10^9^/L，ALC 0.91×10^9^/L，HGB 54 g/L，PLT 163×10^9^/L，ARC 0.0113×10^12^/L。血涂片：淋巴细胞占30％，其中LGL占淋巴细胞的56％。骨髓象：增生活跃，粒系占84.0％，红系占3.5％，巨核细胞大致正常，淋巴细胞占10％，为成熟淋巴细胞。免疫分型（外周血）：T-LGL占淋巴细胞58.22％，TCRvβ13.1亚家族比例明显增高，为87.18％。TCR重排阳性。2019年12月25日予以口服西达本胺30 mg 每周两次治疗，逐渐脱离输血，PLT 波动于（66～163）×10^9^/L，ALC呈下降趋势，随访11周时血常规：WBC 6.53×10^9^/L，HGB 82 g/L，PLT 70×10^9^/L，ANC 5.31×10^9^/L，ALC 0.54×10^9^/L，ARC 0.0589×10^12^/L。随访22周时血常规：WBC 3.89×10^9^/L，HGB 83 g/L，PLT 160×10^9^/L，ANC 3.23×10^9^/L，ALC 0.14×10^9^/L，ARC 0.0887×10^12^/L。6个月后患者因肺感染，停用西达本胺。后未再应用其他治疗，间断输血支持。

例2，女，63岁，因“体检发现淋巴细胞增高2年”于2020年7月31日就诊于我院，患者2年前于当地诊断T-LGLL，初诊血常规：WBC 5×10^9^/L，ALC 3.9×10^9^/L，HGB 94 g/L，PLT 238×10^9^/L。2018年9月予以口服环孢素、安雄治疗，贫血一过性好转后再次下降，2019年12月调整为环磷酰胺100 mg每日1次联合泼尼松20～30 mg每日1次治疗6个月，贫血进一步加重，为进一步诊治来我院。入院查体：贫血貌，浅表淋巴结未扪及肿大，心肺未见异常，肝脾肋缘下未触及。血常规：WBC 4.94×10^9^/L，ANC 2.49×10^9^/L，HGB 82 g/L（输红细胞4 U后），PLT 367×10^9^/L，ARC 0.0112×10^12^/L。贫血四项筛查：SF 209.8 µg/L，EPO>252.82 IU/L，FA及VB12正常。免疫分型（外周血）：CD3^+^/CD4^−^/CD8^+^/CD57^+^/TCRαβ^+^T-LGL占淋巴细胞67.9％，LGL绝对值：1.5×10^9^/L。TCR重排阳性。外周血涂片：淋巴细胞占61％，比例明显增高，不典型淋巴细胞易见，胞体较大、胞质丰富、略嗜碱性、胞质中有数量不等的大嗜天青颗粒，约占40％。骨髓象：增生大致正常，粒系49.5％，红系1.0％，巨核细胞17个，淋巴细胞占44.0％，LGL易见。染色体为正常核型。二代测序：STAT3（c.1842C>G/p.S614R）突变。诊断为T-LGLL；PRCA。2020年8月10日予以口服西达本胺25 mg每周两次，用药1周后复查血常规：WBC 4.12×10^9^/L，HGB 70 g/L（输红细胞2 U后），PLT 250×10^9^/L，ANC 0.39×10^9^/L，ALC 3.43×10^9^/L，ARC 0.0234×10^12^/L，期间出现上呼吸道感染，予以升白细胞、头孢哌酮钠舒巴坦钠抗感染治疗后好转出院。后未再输血，定期监测血常规：ALC波动于（3.73～6.17）×10^9^/L，HGB波动于70～80 g/L，PLT波动于（78～137）×10^9^/L，ANC波动于（0.09～0.33）×10^9^/L，间断注射G-CSF。治疗后3个月血常规：WBC 24.87×10^9^/L，HGB 84 g/L，PLT 137×10^9^/L，ANC 15.34×10^9^/L，ARC 0.0682×10^12^/L。后由于胃肠道不适调整剂量为20 mg每周两次，治疗后6个月血常规: WBC 6.93×10^9^/L，HGB 80 g/L，PLT 78×10^9^/L，ANC 0.3×10^9^/L，ARC 0.0682×10^12^/L。此后患者因腹泻不能耐受而停用西达本胺。

## 讨论

LGLL的发病机制尚未完全清楚，既往普遍认为慢性抗原刺激和基因突变协同作用下产生LGL克隆性增殖，近年来发现表观遗传修饰也参与了LGLL的发病过程。研究发现，HDAC在许多肿瘤中显示出活性，抑制HDAC活性或表达为治疗肿瘤提供了新策略[6]–[8]。组蛋白的乙酰化程度由组蛋白乙酰化酶和HDAC协调控制，HDAC能将核小体核心组蛋白氨基末端的赖氨酸残基中的乙酰基去除，使得染色体发生凝聚，从而阻止转录激活子进入其靶位点，导致基因表达被抑制。目前已经建立了HDAC抑制剂的几种潜在的抗肿瘤机制，包括抑制细胞增殖、诱导细胞凋亡和细胞分化以及抑制血管生成等。此外，HDAC抑制剂可增强NK细胞对肿瘤细胞的杀伤敏感性，增强人免疫细胞介导的细胞毒性作用，同时增加参与NK细胞活性的蛋白质的表达[9]。NK-LGLL是LGLL的一种少见类型，Sun等[10]研究发现HDAC抑制剂伏立诺他（该药已被FDA批准用于治疗皮肤T细胞淋巴瘤）可降低白血病 NK-LGLⅠ类和Ⅱ类HDAC的表达、增强组蛋白乙酰化，并通过抑制ERK和STAT3细胞信号传导途径诱导NK-LGL细胞凋亡，以时间和剂量依赖性方式抑制白血病NK-LGL细胞的增殖活性。另外，文献报道西达本胺可通过调节JAK2/STAT3信号传导抑制骨髓增生异常综合征和急性髓系白血病细胞活性，而JAK-STAT信号通路也是LGLL发病最重要的信号通路[11]。Poh等[12]报道1例应用HDAC抑制剂贝利司他（该药已被FDA批准用于治疗复发难治的侵袭性PTCL）治疗1例复发/难治的T-LGLL，该患者接受贝利司他治疗3个月后脱离输血至今（超过15个月），并且不良反应可耐受。我们尝试应用西达本胺治疗两例难治T-LGLL患者，两例患者在治疗后约2周时即脱离输血，也显示出了非常好的治疗效果，遗憾的是均因药物不耐受而停药。

体外实验发现西达本胺对正常细胞较肿瘤细胞毒性要小得多，在小鼠和人类异种移植模型中发现西达本胺具有剂量依赖的抗肿瘤活性，且没有明显的毒性作用[9]。临床应用中西达本胺显示出较好的安全性，不良事件多为Ⅰ～Ⅱ级 [13]。而本文两例应用西达本胺的患者在治疗期间出现了不同程度的血液学及非血液学不良反应（包括轻度的血小板减少、淋巴细胞减少、粒细胞缺乏及腹泻）并停药。可能与经过多种治疗后患者体质较弱有关，或者仅仅是因为参与研究的患者较少引起的选择偏移，也可能反映了该疾病的独特生物学特性，未来还需要进一步的探索。

总之，LGLL的治疗有其局限性，对于免疫抑制药物治疗无效的LGLL患者的最佳治疗尚未达成共识。在深入了解LGLL发病机制的基础上探索新型治疗方法十分必要。本研究初次探索了西达本胺治疗T-LGLL的疗效及安全性，为临床医师提供治疗经验，其最优剂量、联合用药及不良反应的处理等仍需要进一步的研究探索。
